# Characteristics of Inpatient Hypertension Cases and Factors Associated with Admission Outcomes in Ashanti Region, Ghana: An Analytic Cross-Sectional Study

**DOI:** 10.1155/2017/6537956

**Published:** 2017-12-05

**Authors:** Kenneth Nuamah, Harriet Affran Bonful, Joseph Danso Yeboah, Ebenezer Antwi Amankwaah, Daniel Boakye, Samuel Kwame Owusu, Adwoa Aduako Owusu, Freddie Amponsah, Fred Adomako-Boateng, Alexis Nang-Beifubah, Margaret Gyapong, Anthony Ofosu, Bertha Garshong, Evelyn K. Ansah

**Affiliations:** ^1^Ashanti Regional Health Directorate, P.O. Box 1908, Kumasi, Ghana; ^2^Department of Epidemiology and Disease Control, School of Public Health, University of Ghana, P.O. Box LG 13, Legon, Accra, Ghana; ^3^Sekyere Central District Health Directorate, P.O. Box 36, Nsuta, Ashanti, Ghana; ^4^Suntreso Government Hospital, P.O. Box 14775, Kumasi, Ghana; ^5^Policy Planning Monitoring and Evaluation Division, Ghana Health Service, PMB Ministries, Accra, Ghana; ^6^Research Development Division, Ghana Health Service, P.O. Box MB-190, Accra, Ghana; ^7^Institute of Health Research, University of Health & Allied Science, PMB 31, Ho, Ghana

## Abstract

**Background:**

Hypertension remains a cause of morbidity and mortality in the Ashanti Region of Ghana. It has been featured in the top ten causes of OPD attendance, admissions, and deaths since 2012. We investigated the sociodemographic characteristics and spatial distribution of inpatient hypertensives and factors associated with their admission outcomes.

**Methods:**

A 2014 line list of 1715 inpatient HPT cases aged ≥25 years was used for the cross-sectional analytic study. Accounting for clustering, all analyses were performed using the* “svy”* command in Stata. Frequencies, Chi-square test, and logistic regression analysis were used in the analysis. Arc view Geographic Information System (ArcGIS) was used to map the density of cases by place of residence and reporting hospital.

**Results:**

Mean age of cases was 58 (S.D 0.0068). Females constituted 67.6% of the cases. Age, gender, and NHIS status were significantly associated with admission outcomes. Cases were clustered in the regional capital and bordering districts. However, low case densities were recorded in the latter.

**Conclusion:**

Increasing NHIS access can potentially impact positively on hypertension admission outcomes. Health educational campaigns targeting men are recommended to address hypertension-related issues.

## 1. Introduction

The World Health Organization (WHO) defines hypertension as a systolic blood pressure equal to or above 140 mm Hg and/or diastolic blood pressure equal to or above 90 mm Hg [1]. Overall health and wellbeing of human rely on efficient functioning of vital organs such as the heart, brain, and kidneys [1]. This largely depends on both systolic and diastolic blood pressure being normal. If hypertension is left uncontrolled, it can lead to several cardiovascular complications including a heart attack, an enlargement of the heart, and eventually heart failure [1]. Kidney failure, blindness, rupture of blood vessels, and cognitive impairment can be attributed to hypertension [1].

The prevalence of hypertension rose from 600 million in 1980 to about 1 billion in 2013 [1]. Worldwide, there are over 17 million deaths attributed to cardiovascular diseases, which is nearly one-third of total deaths in a year [2]. Complications due to hypertension account for 9.4 million deaths every year [3]. Hypertension accounts for at least 5% of deaths from heart disease and 51% of deaths due to stroke [3].

The African region has the highest prevalence of hypertension, affecting 46% of all adult aged 25 years and above [4]. The prevalence (35%) in high-income countries is lower compared to 40% in low-income countries. The greatest health challenge facing the African region after HIV/AIDS is hypertension [4].

In sub-Saharan Africa, Noncommunicable Diseases (NCDs) have become important causes of morbidity and mortality, contributing 22% of total deaths [4]. It has been forecasted that, by 2025, the hypertensive population in developing countries will be 125.5 million people, which will correspond to an increase of 68% from 2008 to 2025 [5, 6].

Hypertension disease risk factors include age, race, family history, being overweight or obese, physical inactivity, tobacco use, high-salt diet, too little vitamin D and potassium in diet, excessive alcohol use, stress, and certain chronic conditions [3]. The prevalence of hypertension increases with increasing age. Sidhu and colleagues noted that the highest prevalence is found in ages equal to or above sixty (60 years) followed by ages between fifty and fifty-nine (50–59 years) [7] whilst Opie and Seedat also noted that, at age 65 in rural West Africa, there is a 30%–40% chance of being hypertensive as compared to 50% chance in semiurban areas in West Africa [4].

Others have identified level of education and occupation as factors associated with hypertension [8]. The risk of hypertension can vary in geographical areas depending on environmental conditions and socioeconomic position [9]

Ghana like other middle-income countries is no exception as hypertension is the number three killer today [10]. The prevalence of high blood pressure in Ghana is estimated at 12.9% and 15% for women and men, respectively [10]. The 2014 Demographic Health Survey puts the prevalence of hypertension in Ashanti at 15.3% for women and 18.1% for men, which is higher than the national average [10]. Prevalence of hypertension for Ashanti is highest among males in Ghana and second highest for females [10].

Earlier studies in the Ashanti Region have shown that there is a long-standing perception that hypertension disease is associated with affluence and that it is a disease of the rich and wealthy in society [11]. This perception has led to many people in low-income communities failing to report for medical check-ups and undertake routine exercises and not fully participate in health education programmes [11].

Over the years, some of the steps taken to control the increasing burden of hypertension in the country by the Ghana Health Service (GHS) have included the introduction of the Regenerative Health and Nutrition Program as well as the institutionalization of hypertension clinics in health facilities. In a systematic review by Tagoe and Dake in 2011, it was concluded strongly that more females exhibited healthier life style compared with males after the introduction of the Regenerative Health Policy [12]. Despite these interventions, morbidity and mortality due to hypertension continue to rise in the region. The proportion of hypertension to total OPD attendance increased from 3.8% in 2012 to 5.4% in 2014 [13]. More importantly, the proportion of admissions attributed to hypertension increased from 1.1% in 2010 to 2.4% in 2014. In 2014, hypertension was ranked sixth, third, and fifth, for top ten causes of OPD attendance, cause of admissions and deaths, respectively [13]. A review of literature suggests that there is little knowledge on the local factors driving the increasing burden of the disease and hospitalization. This study describes the characteristics of inpatient hypertension cases; establishes the factors associated with the outcomes of hypertension admissions; and also determines the geospatial distribution of these admissions in the Ashanti Region for the year 2014.

## 2. Materials and Methods

Data collected was used purposely for a cross-sectional analytic study. The study was conducted in Ashanti Region, which is one of the ten administrative regions of Ghana located in the middle belt of the country and covers about 10.2% of the total land surface area. It has an estimated 2014 mid-year population of 5,461,933, constituting 19.8% of the national population (projection from 2010 population and housing census, Ghana Statistical Service). The Komfo Anokye Teaching Hospital (KATH) is the teaching hospital for the region. Health services are delivered in public, private, quasi-, and mission facilities totaling 548. These facilities are comprised of 121 hospitals, 103 health centres, 282 clinics, and 133 CHPS compounds. Inpatient services are rendered in the 121 hospitals for hypertensive patients. Population to health facility ratio is 1 : 9967. Most facilities report on health indicators into the District Health Information Management System (DHIMS2) software; however, the only teaching hospital which also serves as the main referral hospital in the region does not.

This study used secondary data extracted from* DHIMS 2* for the Ashanti Region. DHIMS 2 is a developed software that operates on* DHIS2 (District Health Information Software)* application platform. DHIMS 2 is used by GHS to collect and collate data on health care service provision. DHIMS 2 helps to generate routine data, analyse reports using maps, and graphs and health profiles for indicators across facilities over a period.

The study population included records of all hypertension patients on admission aged between 25 years and above, diagnosed by clinicians within the Ashanti Region and reported in the DHIMS platform.

Diagnosis made in this study was done by prescribers in accordance with the standard Treatment Guidelines of Ghana [14]. Recruitment of study participants was based on diagnosis of hypertension by clinicians providing care in the health facility. Clinician practice is in accordance with the Standard Treatment Guidelines of Ghana and WHO. There is a system of regular supervision by different levels of health managers to ensure that the guidelines and protocols are adhered to. According to the treatment guideline, prescribers are expected to take an average of two or more properly measured blood pressure readings of a patient before diagnosing them of being hypertensive. At the point of call, the patient to be diagnosed is made to assume a relaxed and comfortable position for at least thirty (30) minutes [14]. Blood pressure (BP) of patients is checked with the use of manual mercury sphygmomanometer and a stethoscope. The arm (preferably left arm) is positioned to face the prescriber and a cuff is wrapped around the upper arm with the cuff lower edge about one inch above the antecubital fossa of the patient [14]. The average of the second and third or more outcomes of systolic blood pressure (SBP) and Diastolic Blood Pressure (DBP) within constant time intervals is recorded as the actual BP of the Patient. An adult with persistent SBP equal to or higher than 140 mm Hg and DBP equal to or higher than 90 mm Hg in nondiabetic or SBP equal to or higher than 130 mm Hg and DBP equal to or higher than 80 mm Hg in diabetics is diagnosed as being hypertensive.

Majority (>95%) of the sphygmomanometer used in the health facilities involved in this study are manufactured by Accoson from England; 3M Littmann stethoscopes from USA are also the commonly used stethoscopes in the Ashanti Region. According to the clinical engineering department, apart from the mandatory conformance to standard specifications checks made on the equipment, other factors including availability and accessibility to spare parts from manufacturers and user friendliness of the equipment, influences the choice of the equipment. Health facilities in Ghana procure clinical equipment including the manual mercury sphygmomanometer and the stethoscope with prior approval and in consultation with the clinical engineering department at the regional headquarters of the Ghana Health Service. The regional clinical engineer's approval and recommendation are paramount in the decision-making process to procure clinical equipment according to standard specifications at all levels of health care delivery (i.e., district hospitals, health centres, and polyclinics).

Hypertension-related conditions excluded in the* ICD-10-CM* (International Classification and Coding of Diseases) tabular list of diseases and injuries codes* (I10–I15)* were excluded in this study. Due to incomplete data for previous years, only data for the year 2014 was used. This study employed a cross-sectional analytic design approach.

The data was “svyset” to account for clustering in facilities and all analyses were conducted using the “svy:” command in Stata version 13 S.E (Stata Corporation, College Station, Texas). The data extracted was checked for internal consistency and completeness using simple summary statistics of the selected variables. Patient folder numbers were used as unique identifiers instead of patient names. All duplicate numbers were deleted before analysis. Frequencies were used to describe the characteristics of cases. Where there was evidence of skewness in the data, means were estimated for continuous variables after log transformation.

The independent variables were age, sex, educational level, occupation, place of residence, length of stay, facility ownership type, and District and National Health Insurance status. Educational level was recategorized from 7 levels (none, primary, JHS/middle school, SHS, technical/vocation, tertiary, and unspecified) to 4 levels (no formal education, primary, secondary, and tertiary) by merging primary and JHS, SHS, and technical vocation. Occupation was recoded from 13 to 6 groups by merging occupational status with similar characteristics as follows:* formal employment *(professional), clerical worker;* self-employed,* business person, farmer, hairdresser, and trader;* unemployed,* housewife, retiree, unemployed, and unskilled laborer*; others; student; and unspecified*. Age and length of stay were also recoded into categorical variables with uniform intervals, that is, age (5 groups with 10-year interval from 25 years to 65 and above.) and length of stay in days (5 groups from 1 day to 5 days and above).

The dependent variable, hypertension admission outcome, and a categorical ordinal variable with three categories* (died, discharged, and referred)* were used to perform Chi-square tests of associations with the independent variables. This variable was then recoded into a binary variable* (died and did not die)* by merging the referred and discharged cases. To identify the predictors of inpatient hypertension admission outcomes, univariable and multivariable logistic regression analyses were performed to determine the predictors of inpatient hypertension admission at 95% confidence level and a *p* value of 0.05.

### 2.1. Geospatial Distribution

To generate the spatial distribution of the cases, the data in Ms Excel format was imported into Arc GIS Version 10.2. The shape files for all the districts in the region adopted from the Ghana Survey Department were used to draw thematic maps for the study since the shape files for the actual communities were not available. The communities of residence (addresses) of the cases were aligned with their respective districts for the analysis of the geographical distribution. Ninety-five (95) individual records were deleted from the list because their addresses could not be linked to any district in the region.

## 3. Results and Discussion

The total sample size obtained after data validation was 1,715. However, there were missing data in some variables in the dataset; therefore the sample size varied for the analysis conducted on these variables. The cases reported were seen in 30 hospitals comprising 17 public (government), 10 quasi-/mission facilities, and 3 private health facilities. Majority of the cases (59.3%) were seen in public hospitals.

Females constituted 67.5%. The mean age of these patients was 58.2 (S.D 0.00685) years, ranging from 25 years to 106 years. More than half (57.7%) of the hypertensive patients on admission had no formal education with the rest having been in some form of academic training as shown in Table 1. A proportion of 85.2% (*n* = 1695) accessed health care with the National Health Insurance Scheme (NHIS) card. The mean length of stay on admission was 2 days, ranging from one to ninety-one (1–91) days. 90% of hypertensive patients on admission were engaged in one form of employment or the other. Less than ten percent (7.5%) were not engaged in any form of occupation.

**Figure 1:**
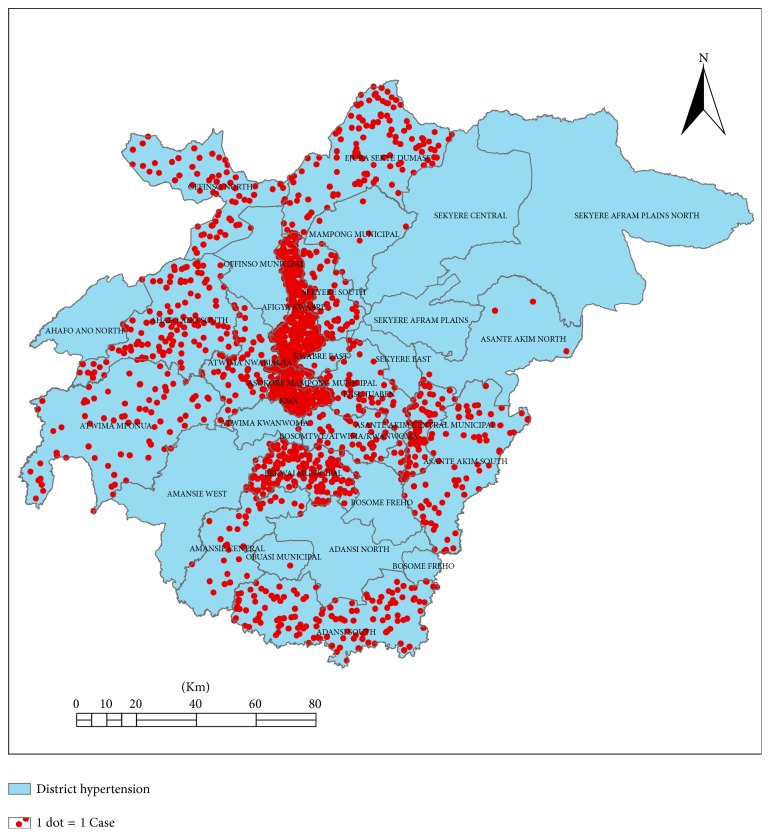
Case density of the hypertension admissions by clients place of residents, Ashanti Region, 2014.

Figure 1 shows the spatial pattern of cases by place of residence (address) across the study area. Most cases were clustered around the regional capital, Kumasi, and adjoining districts, whereas 9 districts including Sekyere Afram plains, Sekyere Central, Sekyere Kumawu, Obuasi Municipal, Ahafo Ano North, Asokore Mampong, and Sekyere East recorded admissions below 5 cases. Four out of these 9 districts do not have district hospitals.

**Figure 2:**
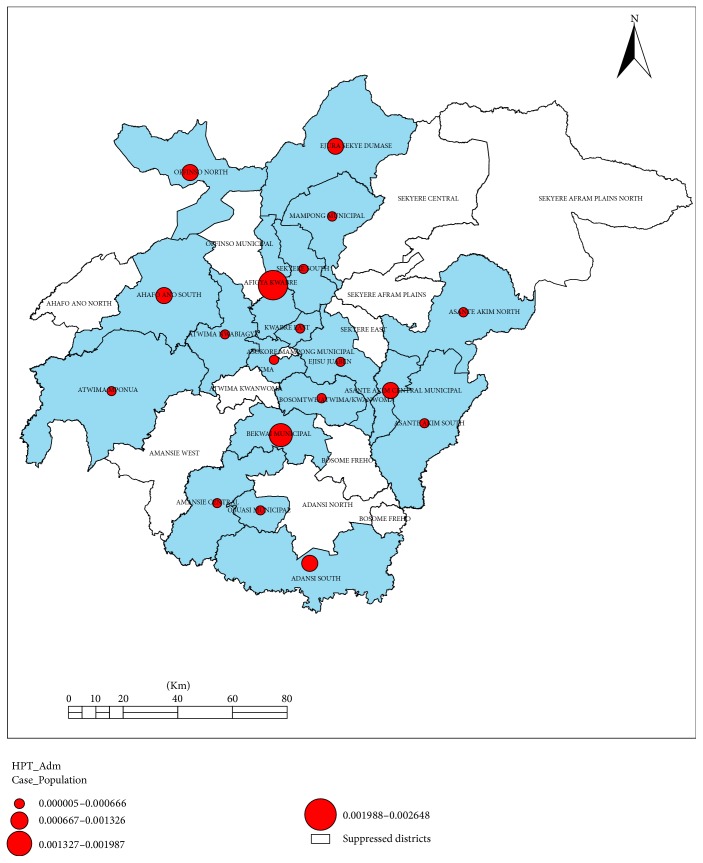
Case density of hypertension admission as reported by hospital, Ashanti, 2014.

Figure 2 is a spatial distribution of hypertension admission case density per hospital. Four (4) hospitals located in 4 districts (Ejura hospital, Nkenkaasu Hospital, Methodist Faith Healing Hospital, and Bekwai Hospital) reported hypertension admission cases constituting more than 0.1% of the district population. These 4 hospitals from 4 districts contributed 9.59% of the total admissions and 14.08% of the total deaths for 2014.

Chi-square tests of association showed no significance between the dependent variable and all independent variables except NHIS (Chi = 10.84, *p* < 0.05) as shown in Table 2.

Crude analysis of the association between the outcome of admission and predictor variables showed that gender, length of stay, and NHIS status were associated with admission outcomes. After adjusting for all other variables, NHIS and sex were significant predictors of the hypertension admission outcome as shown in Table 3.

NHIS registrants were 78% less likely to die from admissions due to hypertension (AOR 0.22, 95% CI 0.11–0.45; *p* = 0.001), whilst females were 51% less likely to die from hypertension admission (AOR 0.49, 95% CI 0.29–0.82; *p* = 0.009).

In the crude analysis, for every day increase in the length of stay on admission, clients were 1.04 times more likely to die from hypertension admission. Replacing age group (categorical variable) with age (continuous variable) and using the same model, age showed a significant association with hypertension admission outcomes (OR = 1.03, 95% CI 1.00–1.05, *p* = 0.029) implying that, for every unit increase in age (years), clients were 1.04 times more likely to die from hypertension admission.

This study sought to use secondary data on hypertension admissions from the DHMIS for 2014 to determine the characteristics of inpatient hypertension cases and factors associated with admission outcomes in Ashanti Region for 2014. Females dominated the number of inpatient hypertensives in this study. This is consistent with Owusu-Sekyere and colleagues who report that there are more female hypertensives in the Kumasi metropolis than males [11]. Our finding is however not consistent with findings of Kolo and colleagues in a similar study, where females constituted 48.4% [15].

In this study, age groups were not associated with admission outcomes. However, age as a continuous variable had significant association with the outcome. Thus, for every unit increase in age (years), hypertensives on admission are 1.04 times more likely to die (OR 1.04, 95% CI 1.003–1.05 *p* = 0.029). This is consistent with Peck et al. study which reported in 2013 that those who die of noncommunicable diseases such as hypertension are older than those who died from communicable diseases [16].

Sex and NHIS status of clients were significantly associated with admission outcomes in this study. Contrary to popular opinions, available data showed that females were 51% less likely to die from hypertension admission. This is probably because women are more conscious of their health with a better health seeking behaviour than men [15]. It can only be inferred that men's health seeking behaviour was poor. There is the need to improve upon the health seeking behaviours of men especially through information, communication, and education programs. Earlier studies by Méndez-Chacón et al. reported that the proportion in men unaware of their hypertension status is higher than that of unaware women (32% versus 20%) [17].

Majority of the deaths recorded from HPT admissions were at the age of 65 years and above with females dominating. However, Williams et al. established the tremendous burden of hypertension-related conditions in adults in sub-Saharan Africa in a similar study which also reported that majority of hypertension-related deaths occurred in the working-age males (median age of death: 61 years) [16].

Our study reports that NHIS registrants are 78% less likely to die from admissions due to hypertension (AOR 0.22, 95% CI 0.11–0.45; *p* = 0.001). Others have reported that the blood pressure levels of clients who receive free medical care are lower than those who have to pay for the cost of care. Therefore, it is plausible that patients who have to pay for cost of care generally delay in seeking prompt care until their conditions are worsened [4]. Obviously, this is likely to lead to a poorer outcome when they are finally admitted. Although the proportion of patients with NHIS clients in this study was high (85.2%), the effects of lack of NHIS ownership on admission outcomes observed in this study bring to bear the need for a universal health care policy, which ensures that primary prevention and management of hypertension are included in the basic health care provided to all citizens.

Earlier reports from Nigeria indicate that case fatality for hypertension admissions is influenced by length of stay in the hospital, among others [15], and this study also answers in the affirmative regarding the crude analysis alone (OR 1.04, 95% CI, *p*- value = 0.009).

Our study shows that majority (59.3%) of the cases were reported by government hospitals. Out of 121 hospitals in the Ashanti Region, hospitals that reported in DHIMS by ownership were 28, 68, and 22 for mission/quasi-, private, and public facilities, respectively. However, the proportion of hospitals that reported on hypertension admission cases for the year 2014 is 35.7% of quasi-/mission, 4.4% of private, and 77.3% of public facilities. This situation contributed to the high number of cases reported by the public hospitals.

## 4. Conclusions

NHIS registrants and females were less likely to die on admission resulting from hypertension; therefore increasing NHIS access can potentially have an impact positively on hypertension admission outcomes. Also, IEC programs should pay more attention to men to help improve their health seeking behaviour, thereby improving hypertension admission outcomes.

Strategies must be adapted to resource quasi-/mission and private facilities in providing hypertension admission care to relieve the pressure on public hospitals.

## 5. Study Limitations

Due to the cross-sectional nature of this dataset, the temporal associations between NHIS status, gender, age, length of stay, and hypertension admission outcome variables may not be causal. Our study did not have any gold standard to compare the hypertension diagnosis provided in the dataset; therefore any misclassification could not have been determined. The actual cause of death could not also be validated due to the nature of the data; therefore, the death reported in this study may not be due to hypertension alone. However, generally, prescribers are all well trained and participate in compulsory continuous professional development programs coupled with regular stringent monitoring and supervisory visit from the Regional Health Administration. It could be presumed that they provide standard care to the patients. The use of routine health facility data restricted this study to the use of only variables presented in the dataset. All the public and quasi-/mission hospitals from the district levels report to the DHIMS platform, but eight out of the thirty districts in the region do not have hospitals to admit HPT cases; therefore cases from these districts could not be reported by the district. The findings of this study may not be generalizable to the whole Ashanti Region granted that even data from the only teaching hospital and biggest referral hospital (KATH) in the region was not available.

## Figures and Tables

**Table 1:** Sociodemographic characteristics of inpatient hypertension clients in Ashanti Region, 2014.

	Female		Male		Total	
	Freq	% (95% CI)	Freq	% (95% CI)	Freq	% (95% CI)
Age group	*N* = 1714					
25–34	61	35.60 (2.79–4.52)	30	1.75 (1.01–3.03)	91	5.31 (3.98–7.05)
35–44	158	9.22 (7.04–11.99)	74	4.32 (3.29–5.64)	232	13.54 (11.55–15.80)
45–54	249	14.53 (12.80–16.44)	132	22.23 (6.03–9.78)	381	22.23 (20.34–24.24)
55–64	229	13.36 (11.73–15.17)	119	6.94 (5.71–8.42)	348	20.3 (18.58–22.14)
65+	460	26.84 (24.51–29.31)	202	11.79 (8.99–15.30)	662	38.62 (36.26–41.05)
Total	1157	67.5 (61.56–72.93)	557	32.50 (27.07–38.44)	1714	100
Sex	*N* = 1714					
Female	1157	67.5 (61.56–72.93)	0	—	1157	67.5 (61.56–72.93)
Male	0	—	557	32.5 (27.07–38.44)	557	32.5 (27.07–38.44)
Total	1157	67.5 (61.56–72.93)	557	32.50 (27.07–38.44)	1714	100
Educational level	*N* = 1713					
No education	706	41.21 (31.31–51.88)	282	16.46 (12.53–21.32)	988	57.68 (45.38–69.09)
Primary	393	22.94 (16.96–30.26)	212	12.38 (8.37–17.92)	605	35.32 (25.92–46.01)
Secondary	33	1.93 (0.73–4.99)	37	2.16 (1.00–4.59)	70	4.09 (1.86–8.74)
Tertiary	24	1.4 (0.67–2.89)	26	1.52 (0.70–3.26)	50	2.92 (1.46–5.75)
Total	1156	67.48 (61.55–72.91)	557	32.52 (27.09–38.45)	1713	100
NHIS status	*N* = 1694					
Not having NHIS	148	8.74 (5.06–14.68)	103	6.08 (3.57–10.17)	251	14.82 (8.82–23.83)
Have NHIS	993	58.62 (50.21–66.55)	450	26.56 (22.69–30.83)	1443	85.18 (76.17–91.18)
Total	1141	67.36 (61.49–72.72)	553	32.64 (27.28–38.51)	1694	100
Occupation	*N* = 1711					
Unemployed	88	5.14 (1.84–13.54)	41	2.40 (12.09–5.20)	129	7.54 (3.02–17.60)
Formal employment	16	0.94 (14.38–43.87)	23	1.34 (0.66–2.73)	39	2.28 (1.23–4.18)
Self-employed	455	26.59 (14.38–43.87)	227	13.27 (7.22–23.13)	682	39.86 (20.67–62.77)
Student	1	5.8*e* − 02 (6.7*e* − 03–0.51)	2	0.12 (2.5*e* − 02–0.54)	3	0.18 (4.8*e* − 02–0.64)
Unspecified	470	27.47 (10.07–56.15)	161	9.41 (3.4–23.48)	631	36.88 (12.52–70.46)
Others	125	7.31 (3.18–15.89)	102	5.96 (2.88–11.93)	227	13.27 (6.41–25.47)
Total	1155	67.50 (61.53–72.96)	556	32.50 (27.04–38.47)	1711	100
Facility ownership		*N* = 1714				
Quasi-/mission	403	23.51 (7.12–55.23)	201	11.73 (4.97–25.22)	604	35.24 (10.8–70.97)
Public	700	40.84 (21.03–64.16)	317	18.49 (9.60–32.66)	1017	59.33 (27.20–85.07)
Private	54	3.15 (0.63–14.29)	39	2.28 (0.56–8.79)	93	5.43 (1.27–20.40)
Total	1157	67.50 (61.56–72.93)	557	32.50 (27.07–38.44)	1714	100
District status	*N* = 1714					
Rural	337	19.66 (8.29–39.84)	200	11.67 (4.87–25.43)	537	31.33 (12.48–59.34)
Urban	820	47.84 (30.18–66.06)	357	20.83 (13.86–30.08)	1177	68.67 (40.66–87.52)
Total	1157	67.5 (61.56–72.93)	557	32.50 (27.07–38.44)	1714	100

**Table 2:** Chi-square test of association of sociodemographic characteristics of inpatient hypertensive clients and outcome of admission.

Variables	Hypertension outcome of admission						Total	*χ* ^2^	*p* – Value
	Died		Discharged		Referred				
	Frequency	%	Frequency	%	Frequency	%			
Age group									
25–34	2	3.9	86	5.39	3	4.5	91	8.82	0.304
35–44	3	5.8	218	13.7	11	16.4	232		
45–54	10	19.2	355	22.3	17	25.4	382		
55–64	8	15.4	326	20.4	14	20.9	348		
65+	29	55.8	610	38.2	22	32.8	661		
*Total*	*52*	*100*	*1595*	*100*	*67*	*100*	*1714*		
Sex									
Male	24	46.2	506	31.7	27	40.3	557	6.69	0.218
Female	28	53.9	1088	68.3	40	59.7	1156		
*Total*	*52*	*100*	*1594*	*100*	*67*	*100*	*1621*		
Educational level									
No formal education	31	59.6	921	57.8	35	52.2	987	7.60	0.414
Primary	16	30.8	564	35.4	25	37.3	605		
Secondary	2	3.9	66	4.1	2	3.0	70		
Tertiary	3	5.8	42	2.6	5	7.5	50		
*Total*	*52*	*100*	*1593*	*100*	*67*	*100*	*1712*		
NHIS status									
Have NHIS	36	69.2	1350	85.7	57	85.1	1443	10.84	0.041^*∗*^
Not having NHIS	16	30.8	225	14.3	10	14.9	251		
*Total*	*52*	*32*	*1575*	*100*	*63*	*100*	*1694*		
Occupation									
Unemployed	3	5.8	113	7.1	13	19.4	129	28.09	0.147
Formal Employment	2	3.9	33	2.1	4	6.0	39		
Self-employed	13	25.0	651	40.9	18	26.9	682		
Student	0	0.0	3	0.2	0	0.0	3		
Unspecified	26	50.0	583	36.6	21	31.3	630		
Others	8	15.4	208	13.1	11	16.4	227		
*Total*	*52*	*100*	*1591*	*100*	*67*	*100*	*1710*		
Length of stay									
1 day	15	34.88	477	31.51	19	33.93	511	11.92	0.155
2 days	7	16.28	473	31.24	12	21.43	492		
3 days	5	11.63	230	15.19	9	16.07	244		
4 days	4	9.30	114	7.53	4	7.14	122		
5+	12	27.91	220	14.53	12	21.43	244		
*Total*	*43*	*100*	*1514*	*100*	*56*	*100*	*1613*		
Facility ownership									
Quasi/mission	25	48.1	556	34.9	23	34.3		8.41	0.706
Public	27	51.9	946	59.3	43	64.2			
Private	0	0	93	5.8	1	1.5			
*Total*	*52*	*100*	*1595*	*100*	*67*	*100*			
District status									
Rural	13	25.00	513	32.16	11	16.42	537	8.41	0.221
Urban	39	75.00	1082	67.84	56	83.58	1177		
*Total*	*52*	*100*	*1595*	*100*	*67*	*100*	*1714*		

^*∗*^Statistically significant.

**Table 3:** Univariable and multivariable logistics regression analysis for the association between outcome of admission and sociodemographic characteristics of inpatient admissions of Ashanti.

Characteristics	*N* ^*∗*^ = 1552			
	Crude OR (95% CI)	*p* value	Adjusted OR (95% CI)	*p* value
Age group				
25–34 (reference)	1			
35–44	0.58	0.475	0.64 (0.13–3.20)	0.573
45–54	1.19	0.836	0.74 (0.05–10.40)	0.815
55–64	1.05	0.947	0.44 (0.06–3.42)	0.420
65+	2.04	0.436	0.54 (0.02–18.13)	0.722
Age^*∗∗*^	1.03	0.029^*∗∗*^	1.04 (0.99–1.10)	0.087
Sex				
Male (reference)	1			
Female	0.55	0.017^*∗∗*^	0.49 (0.29–0.82)	0.009^*∗∗*^
Educational level				
No formal education (reference)	1			
Primary	0.84	0.581	1.43 (0.80–2.56)	0.211
Secondary	0.91	0.905	0.99 (0.13–7.86)	0.999
Tertiary	1.97	0.302	3.42 (0.38–30.49)	0.170
Occupation				
Unemployed (reference)	1			
Formal employment	2.27	0.535	Omitted	
Self employed	0.82	0.746	0.83 (0.14–4.97)	0.834
Student	Omitted		Omitted	
Unspecified	1.81	0.324	2.53 (0.49–13.11)	0.257
Others	1.53	0.533	1.15 (0.19–6.94)	0.872
NHIS status				
Not having NHIS (reference)	1			
Have NHIS	0.376	0.040^*∗∗*^	0.22 (0.11–0.45)	0.001^*∗∗*^
Length of stay on admission				
Length of stay	1.04	0.009^*∗∗*^	1.05 (0.99–1.10)	0.069
Facility ownership				
Quasi/mission (reference)	1			
Public	0.632	0.260	0.69 (0.29–1.61)	0.373
Private	1 (omitted)			
District status				
Rural (reference)	1			
Urban	1.38	0.520	0.84 (0.31–2.30)	0.726

*N* ^*∗*^ sample size for multivariable logistic regression analysis. Age^*∗∗*^ significantly associated with the outcomes in this model when it was substituted for age category.

## Abbreviations

AIDS: Acquired Immune Deficiency Syndrome
AOR: Adjusted odds ratio
CI: Confidence interval
DHIMS: District Health Information Management System
ERC: Ethical Review Committee
GHS: Ghana Health Service
HIV: Human Immune Deficiency Virus
HPT: Hypertension
JHS: Junior High School
KATH: Komfo Anokye Teaching Hospital
MOH: Ministry of Health
NCD: Noncommunicable diseases
NHIS: National Health Insurance Scheme
OR: Odds ratio
OPD: Out-patient department
RHMT: Regional Health Management Team
WHO: World Health Organization.
